# A detailed genome-wide reconstruction of mouse metabolism based on human Recon 1

**DOI:** 10.1186/1752-0509-4-140

**Published:** 2010-10-19

**Authors:** Martin I Sigurdsson, Neema Jamshidi, Eirikur Steingrimsson, Ines Thiele, Bernhard Ø Palsson

**Affiliations:** 1 Department of Biochemistry and Molecular Biology, Faculty of Medicine, University of Iceland, Reykjavik, Iceland; 2 Department of Genetics and Molecular Medicine, Landspitali-University Hospital, Reykjavik, Iceland; 3 Center for Systems Biology, University of Iceland, Reykjavik, Iceland; 4 Department of Bioengineering, University of California, San Diego, La Jolla, California 92093-0412, USA; 5 Faculty of Industrial Engineering, Mechanical Engineering & Computer Science, University of Iceland, Reykjavik, Iceland

## Abstract

**Background:**

Well-curated and validated network reconstructions are extremely valuable tools in systems biology. Detailed metabolic reconstructions of mammals have recently emerged, including human reconstructions. They raise the question if the various successful applications of microbial reconstructions can be replicated in complex organisms.

**Results:**

We mapped the published, detailed reconstruction of human metabolism (Recon 1) to other mammals. By searching for genes homologous to Recon 1 genes within mammalian genomes, we were able to create draft metabolic reconstructions of five mammals, including the mouse. Each draft reconstruction was created in compartmentalized and non-compartmentalized version via two different approaches. Using gap-filling algorithms, we were able to produce all cellular components with three out of four versions of the mouse metabolic reconstruction. We finalized a functional model by iterative testing until it passed a predefined set of 260 validation tests. The reconstruction is the largest, most comprehensive mouse reconstruction to-date, accounting for 1,415 genes coding for 2,212 gene-associated reactions and 1,514 non-gene-associated reactions.

We tested the mouse model for phenotype prediction capabilities. The majority of predicted essential genes were also essential in vivo. However, our non-tissue specific model was unable to predict gene essentiality for many of the metabolic genes shown to be essential in vivo. Our knockout simulation of the lipoprotein lipase gene correlated well with experimental results, suggesting that softer phenotypes can also be simulated.

**Conclusions:**

We have created a high-quality mouse genome-scale metabolic reconstruction, iMM1415 (*Mus Musculus*, 1415 genes). We demonstrate that the mouse model can be used to perform phenotype simulations, similar to models of microbe metabolism. Since the mouse is an important experimental organism, this model should become an essential tool for studying metabolic phenotypes in mice, including outcomes from drug screening.

## Background

The first genome-scale reconstruction of metabolic networks emerged eleven years ago [1], four years after the first whole genome sequencing of an entire organism was published [2]. To date, 29 bacteria, 2 archaea and 5 eukaryotes have been reconstructed and for some organisms, up to 5 updates have been published [3]. The reconstruction process is well established for metabolic networks [4]. Once assembled, the reconstruction can be readily converted into a mathematical format by adding balances (e.g., mass -balance constraints), steady-state assumptions and bounds (e.g. physical constraints) [5]. The resulting model is condition-specific and can be used for phenotype simulations using various constraint-based reconstruction and analysis (COBRA) methods [5,6]. This approach has proven successful for various microorganisms and eukaryotes for addressing various biological and biotechnological questions, such as the analysis of knowledge gaps [7], simulation of phenotype traits [8], analysis of evolution of metabolic networks [9,10] and metabolic engineering applications [11]. The numerous applications have recently been reviewed [3,12].

The release of the human genome [13] and its annotation has provided the appropriate foundation for human metabolic reconstructions. Three approaches have been published to date. Two of those, the HumanCyc [14] and the Edinburgh Human Metabolic Network [15,16] were created with a largely automated top-down approach, while Recon 1 [17] was created by an extensive bottom-up manual curation of an automated draft map, including extensive gap-assessment and filling using bibliomic data. Recon 1 has eight compartments (cytoplasm, extracellular space, mitochondria, Golgi apparatus, endoplasmic reticulum, lysosome, peroxisome and nucleus) and accounts for 1496 genes coding for 2004 proteins [17]. Overall, Recon 1 covers all major metabolic pathways occurring in any human cell by accounting for 2,233 biochemical transformations and 1,510 transport and exchange reactions. The Recon 1 can be converted into a functional *in silico *model can be converted into a functional model of human metabolism, which was validated by applying 288 flux balance analysis (FBA) based tests of core functionality of human metabolism [17]. A recent use of Recon 1 includes a study on the topology of human disease finding that several co-morbid diseases had a functional relationship within Recon 1 [18]. Also, expression profiling data mapped onto Recon 1 was used to create a cellular network for ten human tissue types [19]. Furthermore a systems based analysis of the effects of imprinted genes on metabolic functions has recently been published [20].

Metabolic networks have been reconstructed for two further mammals, a central metabolic reconstruction of cattle (*Bos taurus*) [21] and four mouse (*Mus musculus) *reconstructions [22-25].Two of these mouse reconstructions were constructed manually [24,25], while the remaining two were generated semi-automatically [22,23]. The first genome-scale manual curated reconstruction represents the metabolism of mouse hybridoma cell lines [25] and has recently been expanded to represent 1494 reactions coded by 724 genes in three compartments [24]. In addition, a pre-genome sequencing reaction network of intermediate metabolism in mouse hybridoma cell lines was built and analyzed using linear optimization [26].

The mouse serves as a fundamental experimental animal for human biomedical applications. Furthermore, the availability of phenotyped inbred knockout mouse strains [27] make it ideal for examining the phenotype prediction capabilities of mammalian reconstructions. Therefore, a detailed reconstruction of mouse metabolism that can be converted into highly functional model is of a great importance.

Given the high sequence homology between most mammalian genomes, an obvious starting point for metabolic reconstruction of mammals is the comprehensive human metabolic reconstruction, Recon 1. We therefore sought to create draft mammalian metabolic reconstructions based on Recon 1. The mouse reconstruction was then manually completed and validated. We also present simulation results using a functional model of mouse metabolism to test its phenotype prediction capabilities.

## Results

### Identification of homologous genes

The HomoloGene database contains information on homologous genes in 5 mammals and 15 non-mammals [28]. We searched the HomoloGene database for all 1496 genes of Recon 1 and found a human match for 1,464 genes (97.8%). The mammalian organism with the highest number of genes homologous to Recon 1 genes was the mouse (*Mus musculus*) (1,415 genes, 97%). The non-mammalian organism with the highest number of genes homologous to Recon 1 genes was the zebra-fish (*Danio rerio) *(1,200 genes, 82%) (Additional file 1).

### Creation of draft metabolic network reconstructions

**Figure 1:**
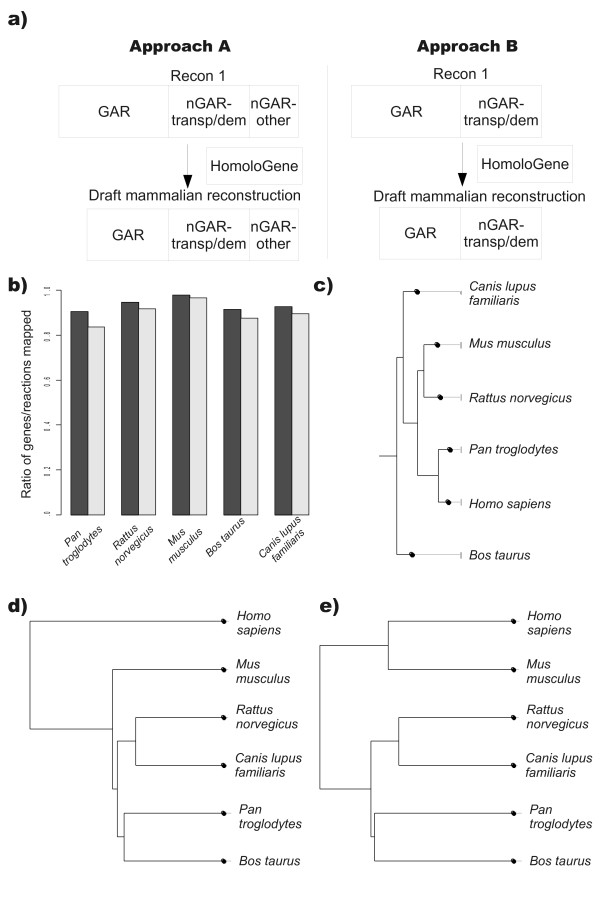
**Creation of draft mammalian reconstructions**. **a) **A schematic figure showing the two approaches used to generate draft mammalian reconstructions using Recon 1. Approach A removes all gene-associated reactions from Recon 1 without a homologous gene in the reconstructed animal, while keeping all non-gene-associated reactions. Approach B removes all gene-associated reactions from Recon 1 without a homologous gene in the reconstructed as well as non-gene-associated reactions (excluding transporters and demand reactions). GAR - gene-associated-reactions; nGAR - non-gene-associated reaction; transp/dem - transporters and demand reactions. **b) **Ratio of reactions (black bar) and genes (gray bar) that were successfully mapped from Recon 1 to the indicated mammalian draft reconstruction. **c) **A phylogenetic tree based on all transcripts of protein domain sequences from the SuperFamily database [64] for all reconstructed mammals. **d) **A phylogenetic tree based on flux variability analysis (FVA) of all reactions in all mammals reconstructed via approach A. **e) **A phylogenetic tree based on flux variability analysis (FVA) of all reactions in all mammals reconstructed via approach B.

For each mammal, a draft reconstruction was created via two different approaches (Figure 1a). In approach A (modelA), all reactions linked to Recon 1 genes that did not have a homologous gene were removed from the reconstruction of the corresponding species. Therefore, modelA included all reactions linked to genes homologous to Recon 1 genes in addition to all non-gene associated reactions (1,514). Of the non-gene-associated reactions, 676 were transporter reactions, 452 were demand or exchange reactions, leaving 385 reactions within a metabolic pathway but without gene-association. In approach B (modelB), we included only genes and their reactions homologues to Recon 1 genes as well as transporter and demand reactions. All other reactions, including non-gene associated reactions, were removed from the reconstruction.

The ratio of Recon 1 genes with a homologous gene as well as the ratio of reactions from Recon 1 included in the draft reconstructions for each mammal is shown in Figure 1b. The mammal with the highest ratio of mapped genes (97%) and reactions (98%) was the mouse while the chimpanzee (*Pan troglodytes*) had the lowest ratio of both mapped genes (84%) and reactions (91%). Furthermore, we created for each mammalian modelA and modelB a compartmentalized and non-compartmentalized version. The more complex, compartmentalized versions had more metabolic dead ends (321 vs. 150) and therefore more reactions with zero fluxes. The number of reactions with non-zero fluxes for both compartmentalized and non-compartmentalized versions of all draft mammalian models created using approach A and B are summarized in Table 1. We performed flux variability analysis (FVA) of models of these reconstructions, allowing uptake of all extracellular metabolites in all models in order to maximize likelihood of non-zero fluxes. Compared to humans, the mouse model had the highest percentage (84-89%) and the chimpanzee model had the lowest percentage (41-54%) of active reactions with non-zero fluxes for all model versions (Table 1). We then constructed a phylogenetic tree of the reconstructed mammals based on the FVA results (Figure 1d,e). The results indicated that the mouse draft model had the highest degree of similarity with the human model for both modelA (Figure 1d) and modelB (Figure 1e). These findings are surprising as the chimpanzee is the closest relative of humans with approximately 99% overall genome similarity [29]. It is also the closest ancestor to humans of the mammals reconstructed when protein coding sequence is compared (Figure 1c).

**Table 1:** Results from flux variability analysis of the draft mammalian models compared to the human model (H. sapiens, Recon 1).

	Approach A				Approach B			
	
	Compartm.		Non-Compartm.		Compartm.		Non-Compartm.	
	
Organism	*N*	*%*	*N*	*%*	*N*	*%*	*N*	*%*
H. sapiens	3189		2037		957		1280	
P. troglodytes	1566	49%	1074	53%	394	41%	688	54%
R. norvegius	2113	66%	1503	74%	726	76%	911	71%
M. musculus	2753	86%	1720	84%	851	89%	1127	88%
B. taurus	1716	54%	1129	55%	556	58%	787	61%
C. lupus fam.	1971	62%	1249	61%	627	66%	842	66%

Absolute number (N) and percentage (%) of active reactions (with at least one non-zero flux) for both compartmentalized and non-compartmentalized models created using approach A and B.

### Identification of unique metabolic functions in the mouse

Given the high number of reactions mapped to the draft mouse reconstruction, and the high number of reactions with non-zero fluxes resulting in the corresponding metabolic modelA and modelB, we sought to finalize the mouse reconstruction by gap analysis/filling. First, we aimed to understand the metabolic differences between human and mouse to ensure that the mouse reconstruction is not merely a modified version of human metabolism. This implies that also genes and pathways unique to mouse metabolism needed to be identified. Therefore, we employed the Comparative Pathway Analyzer 1.0 [30] relying on the KEGG database [31] to extract metabolic maps displaying the existence of enzymes in both mouse and human for 66 out of 99 Recon 1 subsystems (Additional file 2). We then used these maps to perform a manual search of potential gaps between human and mouse metabolism. Out of 1550 reactions present within these subsystems, 1492 (96%) reactions existed in both species, 46 (3%) reactions existed only in humans and 1 (1%) reactions existed only in the mouse (Additional file 2). For the 25 remaining Recon 1 subsystems (excluding transporters), we did an extensive literature search for differences between the two species. Of those, only cytochrome P 450 metabolism was reported to differ significantly between mouse and human [32]. These defined differences will guide the subsequent gap filling process. However, since most of the metabolic functions seemed to be present in both human and mouse, we can employ the validation tests which were designed to evaluate the predictive potential of Recon 1 [17].

### Gap filling of the mouse metabolic reconstruction

First, we decided to test which of the four mouse metabolic models were able to produce biomass by optimizing for the corresponding reaction accounting for all known biomass precursors required for cell replication. The compartmentalized and non-compartmentalized draft mouse models from approach A produced biomass without any modifications. In contrast, both versions of modelB were unable to produce biomass. Therefore, we sought to gap fill the draft non-compartmentalized modelB to identify reactions that needed to be added to the model for production of all biomass components. The SMILEY gap filling algorithm identifies reactions from a database that need to be added to a model to fulfill the optimality condition (e.g. production of biomass) [7].^. ^Using the SMILEY algorithm, we searched for the minimal number of reactions within the entire KEGG database necessary to add to the non-compartmentalized mouse modelB in order to enable the production of biomass.

The results from 40 iterations of the algorithm were checked manually for two criteria: i) the result did not suggest a reversible reaction for a known irreversible reaction and ii) the added reaction(s) are known to occur in mouse. Of the 40 iterations, 30 suggested adding the same reaction either alone or in addition to some other metabolic reactions. This reaction (KEGG ID R06522) exists in mouse and humans (according to KEGG and Entrez gene [33]) but was not included in Recon1. It is a phosphohydrolase reaction involved in sphingolipid metabolism. Adding this reaction resulted in the ability of the refined non-compartmentalized modelB model to produce biomass at a similar rate as the non-compartmentalized modelA. The addition did, however, not result in biomass production in the compartmentalized modelB. Subsequently, we decided to focus the remainder of the study on modelA, as it captures most of the known metabolic capabilities in the mouse. ModelB was not further developed since it is missing a significant fraction of metabolic reactions and therefore does not function.

### Validation of the mouse metabolic reconstruction

To validate the mouse reconstruction and to ensure mouse-characteristic metabolic properties of the resulting models, we manually tested each of the 288 FBA validation tests that were developed for Recon 1 [17] using both versions of modelA. Our literature survey on mouse metabolism, also focusing on the mouse essentiality of the 288 human tests, revealed no tests that were essential only in the human metabolism. Furthermore we found no evidence of mouse-specific additional tests of essentiality that could be added to our validation process. Therefore, we evaluated manually if all required reactions for each test are present in the mouse metabolism using Comparative Pathway Analyzer 1.0. A total of 260 tests passed the requirements and were therefore used for validation of the compartmentalized mouse modelA (Additional file 3). For the non-compartmentalized version of modelA, six additional tests were removed since they were compartment specific, resulting in 254 validation tests.

We ran the validation tests on the compartmentalized mouse draft modelA in an iterative manner and evaluated particularly the failed tests. This process revealed that out of the 131 reactions initially removed due to missing homologous genes in the HomoloGene database, 36 had sequence and physiological evidence of existence in the mouse (according to KEGG and EntrezGene databases) (Additional file 4). Those 36 reactions were therefore added again to the model, but the remaining 95 reactions were left out of the final model due to missing physiologic or sequence evidence. This addition resulted in a finalized mouse modelA that passed all the 260 validation tests. Also, we added the reaction discovered by the gap filling of modelB (KEGG ID R06522). Furthermore, unique mouse reactions which do not lead to metabolic dead ends in the model were added (KEGG IDs R03184, R00647 and R01465). The non-compartmentalized modelA similarly passed 100% of its 254 validation tests after the addition of these reactions. We also determined the functionality of modelB, even though the compartmentalized version cannot produce biomass. The compartmentalized and non-compartmentalized models created via approach B passed 50% and 85% of the validation tests respectively.

### Properties of iMM1415

The resulting compartmentalized mouse metabolic reconstruction (Additional file 5), created by curation of modelA, termed iMM1415, contains a total of 3,724 reactions and 1,415 metabolic genes (Additional file 6 contains detailed description of all reactions in iMM1415, including the biomass reaction). Analysis of the reactions removed from Recon 1 during the creation of iMM1415 revealed that the greatest absolute number of non-included reactions in the mouse reconstruction were within steroid metabolism (16 out of 46 reactions) and blood group biosynthesis (14 out of 46 reactions). However, the largest percentage of non-included reactions was within the subsystems of stilbene, coumarine and lignin biosynthesis (50%, 1 out of 2 reactions) and limonene and pinene degradation (50%, 3 out of 6 reactions). This data suggests that simulation results from these metabolism subsystems will be more likely to be inaccurate and points out areas for future improvement of the mouse model. In the remainder of the paper, we will use models derived from iMM1415 for computations.

### Comparison of iMM1415 with published mouse metabolic networks

Table 2 provides a comparison between our reconstruction and other published reconstructions, the most detailed to date being the recently published mouse metabolic network by Selvarasu *et al *[24]. Our model functions in eight cellular compartments compared to three compartments in most reconstructions. It has the most detailed description of mitochondria metabolism to date but fewer reactions in the cytosol. Furthermore iMM1415 contains the first attempt to describe metabolism in the golgi apparatus, lysosome, ribosome, peroxisome and nucleus. Due to the compartmentalization, a much greater number of transport reactions was required in our reconstruction compared to the previous ones. As less biochemical data exists on transport reactions a larger number of non-gene associated reactions are needed in our model than in the other models. Albeit difficult to compare directly due to different definition of minimum growth medium and biomass, our model predicts fewer essential genes but a higher percentage of them have been experimentally verified than in the other models published to date (Table 2).

**Table 2:** Properties of iMM1415 and comparison with existing models

	iMM1415	**Selvarasu *et al *(2010)**.	**Evsikov *et al *(2009)**.	**Quek *et al *(2008)**.	**Sheikh *et al *(2005)**.
Dominant reconstruction method	Manual	Manual	Automatic	Automatic	Manual
Genes	1,415	724	1,060	1,399	473
Reactions	3,724	715	2,018	1,757	0
Compounds	2,774	1,285	1,377	2,104	872
Compartments	8	3	1	3	3
Reactions	3,726	1,494	2,018	2,037	1,220
Demand& Exchange& Biomass reactions	455	0	0	0	0
Cytosol	931	1,085	NA	1,650	618
Mitochondrial	409	161	NA	387	12
Extracellular	473	NA	NA	NA	NA
Golgi	250	NA	NA	NA	NA
Lysosome	194	NA	NA	NA	NA
Ribosome	184	NA	NA	NA	NA
Peroxisome	95	NA	NA	NA	NA
Nucleus	88	NA	NA	NA	NA
Transport	1,101	248	0	64	267
Non-gene associated reactions	1,514	291	0	148	324
Essential genes according to model^a^	53^a^	109^a^	NA	NA	72
Of which experimental data available	17	20	NA	NA	NA
% match to experimental data	82%	70%	NA	NA	NA
Produces biomass	1	1	0	1	1

^a^under "minimum growth" medium, differences exist in both biomass and minimum growth medium definitions between publications.

NA - not available

### Essentiality of mouse metabolic genes

Given the high degree of functionality of iMM1415 we decided to employ it for phenotype simulations and to compare the *in silico *results to published experimental results from knockout mice or mice with mutations in metabolic genes. First, we performed a simulation of single gene knockouts for all genes in iMM1415 in order to determine *in silico *gene essentiality. A minimal growth medium supplemented with glucose was used for this simulation (as defined in additional file 7). A total of 53 genes were found to be essential as their deletion resulted in zero biomass production (Additional file 8). We found information on homozygous knockout phenotype for 17 of those genes in literature (Table 3). Of those, 14 (82%) genes had a confirmed lethal phenotype, whereas the remaining three (18%) had non-lethal phenotypes (Table 3). The majority of the genes with a predicted and confirmed essentiality status were within the cholesterol metabolism. This result indicates that cholesterol metabolism is especially vulnerable to mutations and environmental insults, perhaps due to the inability of the cholesterol metabolism to overcome disruption in metabolic reactions via alternative pathways.

**Table 3:** Results on gene essentiality predictions by the finalized mouse model.

Gene Name	Reactions	Subgroup	Mutation lethal	Comment	Reference
EBP	3-beta-hydroxysteroid-delta(8),delta(7)-isomerase	Cholesterol Metabolism	yes	X-linked genes, homozygous males are non-viable (prenatal lethality)	Means et al.
DHCR7	7-dehydrocholesterol reductase	Cholesterol Metabolism	yes	Prenatal lethality of homozygotes	Yu et al.
DHCR24	24-dehydrocholesterol reductase	Cholesterol Metabolism	yes	Prenatal lethality of homozygotes	Mirza et al.
FDFT1	Squalene synthase	Cholesterol Metabolism	yes	Prenatal lethality of homozygotes	Tozawa et al.
HSD17B4	C-3 sterol keto reductase, Beta oxidation of long chain fatty acid, 3-hydroxyacyl-CoA dehydrogenase, hydroxysteroid (17-beta) dehydrogenase 4, peroxisomal lumped long chain fatty acid oxidation	Cholesterol Metabolism	yes	Pre/Peri/Postnathal lethality of homozygotes	Huyghe et al.
NSDHL	C-3 sterol dehydrogenase, C-4 methyl sterol oxidase	Cholesterol Metabolism	yes	X-linked gene, males and homozygous females are non-viable (prenatal lethality)	Cunningham et al
SC5DL	Lathosterol oxidase	Cholesterol Metabolism	yes	Perinatal lethality of homozygotes	Krakowiak et al.
SPTLC1	serine palmitoyltransferase, long chain base subunit 1	Sphingolipid Metabolism	yes	Embryonic lethality of homozygotes	Hojjati et al.
DHFR	dihydrofolate reductase, folate reductase	Folate Metabolism	yes	Embryonic lethality of homozygotes	Di Pietro et al.
PISD	phosphatidylserine decarboxylase, mitochondrial	Glycerophospholipid Metabolism	yes	Embryonic lethality of homozygotes	Steenbergen et al.
PHGDH	phosphoglycerate dehydrogenase	Glycine, Serine, and Threonine Metabolism	yes	Embryonic lethality of homozygotes	Yoshida et al.
HMGCR	Hydroxymethylglutaryl CoA reductase (ir)	Cholesterol Metabolism	yes	Embryonic lethality of homozygotes	Tanaka et al.
CBS	cystathionine beta-synthase, selanocystathionine beta-synthase	Methionine Metabolism	yes	Homozygous mice die within 5 weeks after birth	Watanabe et al.
SPTLC2	serine C-palmitoyltransferase	Sphingolipid Metabolism	yes	Embryonic lethality of homozygotes	Hojjati et al.
PAH	L-Phenylalanine,tetrahydrobiopterin:oxygen oxidoreductase	Tyr, Phe, Trp Biosynthesis	no	Homozygous mice with dysruptions in the gene are viable	http://www.informatics.jax.org
TM7SF2	C-14 sterol reductase	Cholesterol Metabolism	no	Although a mixture of mutations can be lethal, homozygotes for mutations are viable	http://www.informatics.jax.org
Gpam	glycerol-3-phosphate acyltransferase	Triacylglycerol Synthesis	no	Homozygous mice are viable	Howerton et al.

Second, we searched the Mouse Genome Informatics database [27] for mouse reconstruction genes where a phenotype had been described with the word "lethality". Furthermore, we required that these genes were homozygous for a gene knockout and had either an embryonic or prenatal lethal phenotype. A total of 88 genes were identified this way. Five genes overlapped with the *in silico *essential genes (*FDFT1, SPTLC1, PHGDH, HMGCR, CBS, SPTLC2*). The observed discrepancy in lethality can be explained by either, i) growth environment simulations, ii) incomplete biomass reaction, iii) missing regulation, iv) wrongly included reactions, or v) any combination of the aforementioned possibilities. Wrongly included reactions could be identified by systematically eliminating non-gene-associated reactions from iMM1415 (e.g., using GrowMatch algorithm [34]) to improve the prediction of lethality. In contrast, the two genes that were essential *in silico *but *in vivo *non-essential for growth suggest missing functions in the metabolic network. Using SMILEY, or related algorithms, it might be possible to identify missing candidate genes.

### Prediction of normal phenotypes

To further evaluate the predictive potential of iMM1415, we identified genes from the Mouse Genome Informatics database [27], for which a null mutation resulted in a normal phenotype. A total of eight such genes were found that were also present in the mouse reconstruction *(OCRL, ACO1, PAFAH1B3, PGM1, FUT9, RHBG, ITPKC, SORD). *For five of those, a gene deletion had no effect in the model, since isozymes existed within the reconstruction. For the three remaining genes (*PGM1, FUT9, SORD*), the deletion led to elimination of corresponding reactions. The effect of these deletions on growth depends on i) the presence of alternative reactions/pathways or ii) importance of reactions for biomass precursor synthesis. We investigated the effect of these three gene deletions on the overall network by performing a FVA. We then compared the FVA results of essential gene knockout (*DHCR7*) to the FVA results of wild type (Table 4) by considering only major sections of metabolism (Table 4). The largest perturbation resulted from the knockout simulation of the essential gene *DHCR7*, where seven major subsections of metabolism changed significantly. The knockout simulation of *PGM1 *led to significant changes within six major subsections of metabolism. The knockout simulation of the *FUT9 *gene led to significant changes within two major subsections of metabolism and the knockout simulation of *SORD *led to no significant changes within the major subsections of metabolism. Therefore, we suggest that six out of the eight genes are not likely to have noticeable metabolic phenotypes under our minimal medium condition due to presence of isozymes or alternative reactions/pathways.

**Table 4:** Results from Flux Variability Analysis (FVA) of 3 knockout models for genes with a confirmed normal phenotype (*PGM1, FUT9, SORD*) and one essential gene (*DHCR7*).

	PGM1			FUT9			SORD			DHCR7		
	down	up	p-value	down	up	p-value	down	up	p-value	down	up	p-value
Amino Acid Metabolism	61	160	<0.00001	85	133	0.00115	122	98	0.10560	68	152	<0.00001
Carbohydrate Metabolism	35	55	0.03501	48	48	1.00000	40	55	0.12380	22	75	<0.00001
Cofactor and Vitamin Metabolism	20	75	<0.00001	53	45	0.41900	60	37	0.01953	25	71	<0.00001
Energy Metabolism	12	34	0.001180	19	27	0.23820	27	19	0.23820	13	32	0.00462
Glycan Metabolism	60	283	<0.00001	118	229	<0.00001	166	182	0.39110	49	294	<0.00001
Lipid Metabolism	109	243	<0.00001	165	189	0.20210	196	162	0.07234	128	225	<0.00001
Nucleotide Metabolism	64	103	0.00255	80	91	0.40020	87	90	0.82160	45	122	<0.00001
Other Amino Acids Metabolism	6	19	0.00932	11	14	0.54850	12	13	0.84150	7	18	0.02781
Secondary Metabolites Metabolism	0	0	1.00000	0	0	1.00000	0	0	1.00000	0	0	1.00000
Transporters	368	437	0.01502	397	422	0.38240	393	443	0.08376	337	479	<0.00001

For each reaction within the model, FVA determines the minimum and maximum flux resulting in the optimal solution for the objective function (here: biomass production). Only those solutions that resulted in the same maximum biomass yield as the wild type model were used for the analysis. By comparing the flux range (the span between v_max,i _and v_min,i _for each reaction i) between the knockout model and a wild type model, each reaction was assigned a status of decreased (down), non-changing or increased (up) flux capacity (representing metabolic activity). For each metabolism subsystem, the number of reactions with decreased and increased flux capacity are shown. Significance testing was done by performing a single value Chi-Square test with 1 degree of freedom comparing against even probability of increased and decreased flux capacity. Significant *p*-values (p < 0.05/10) are typeset in bold.

### Analysis of lipoprotein lipase deficiency on *in silico *phenotype

Finally, we sought to simulate knockouts of genes with softer phenotypes. The *LPL *gene encodes lipoprotein lipase (EC 3.1.1.34) [35], an enzyme that hydrolyzes chylomicrons and very low-density lipoproteins (VLDL) into free fatty acids. Individuals born with lipoprotein lipase deficiency have elevated levels of triglycerides and VLDL and suffer from recurrent episodes of abdominal pain and pancreatitis as well as eruptive xanthomas of the skin [35]. Mutations have been associated with increased risk of ischemic heart disease in man [36]. Mice without lipoprotein lipase are born with greatly elevated levels of triglycerides and VLDL, and after nursing, triglyceride levels soon become extremely high. Heterozygotes for the null mutation of *LPL *survive until adulthood but with elevated triglyceride levels [37].

**Figure 2:**
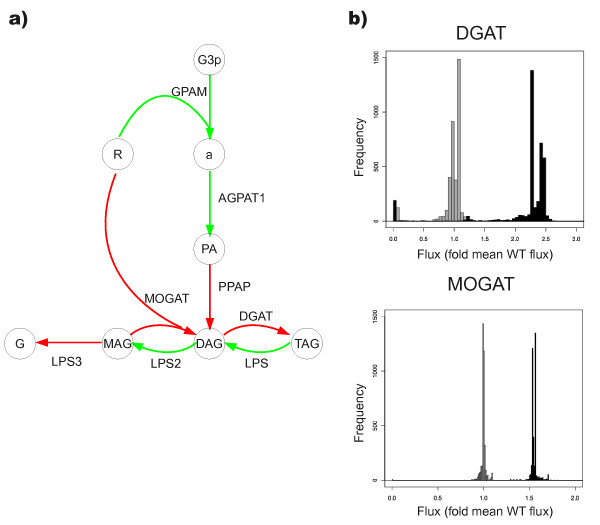
**Results from comparison of the flux variability analysis (FVA) for a LPL knockout model with a wild type model. **Results from comparison of the flux variability analysis (FVA) for a LPL knockout model with a wild type model. a) A part of triacylglycerol metabolism is shown. Reactions with increased flux capacity in the knockout model are shown in red and reactions with decreased flux capacity are shown in green. **b) **The distribution of fluxes in FVA of a wild type model (dark gray) compared to knockout model (black) indicates increased flux through the diacylglycerol acetyltransferase (DGAT) and monoacylglycerol acetyltransferase (MOGAT). These results suggest that hypertriglyceridemia can result from knockout of the LPL gene. Reactions: AGPAT1 - 1-acylglycerol-3-phosphate O-acyltransferase 1; DGAT - diacylglycerol acyltransferase; GPAM - glycerol-3-phosphate acyltransferase; LPS - lipoprotein lipase LPS2 - lipoprotein lipase 2; LPS3 - lipase; MOGAT - monoacylglycerol acyltransferase; PPAP - phosphatidic acid phosphatase. Metabolites: a-lysophosphatidic acid; DAG - diacylglycerol (*Homo sapiens*); G - glycerol; G3p - Glycerol 3-phosphate; MAG - monoacylglycerol 2; PA - phosphatidic acid; R - R groups (total); TAG - triacylglycerol.

We simulated the knockout of the LPL gene and performed FVA to determine the effect of the gene deletion to the network properties. The largest perturbation was observed within i) the glycan metabolism, where 31 reactions had decreased and 312 reactions increased flux capacity (p < 10^-16 ^against even probability of decreased and increased flux capacity); and ii) lipid metabolism, where 100 reactions with decreased and 253 reactions with increased flux capacity (p < 10^-15 ^against even probability of decreased and increased flux capacity). Additionally, the flux capacity changed significantly within the triacylglycerol metabolism (Figure 2). The results suggest that there is increased flux capacity towards triglyceride synthesis while there is decreased flux capacity towards triglyceride degradation. The simulation results are therefore consistent with hypertriglyceridemia resulting from LPL mutations, as previously demonstrated with the mouse knockout model [37-51].

## Discussion

Can the success of metabolic systems biology in microbes be repeated for more complex organisms? A prerequisite for such success is the existence of high quality metabolic network reconstructions. Here, we examined if the detailed and validated network reconstruction of human metabolism, Recon 1, could be used as a basis for reconstructing metabolic networks in other mammals. We then focused on mouse to create a manual curated, comprehensive reconstruction of mouse metabolism that was subsequently employed to determine normal and lethal growth phenotypes. Overall, we found good agreement between the model's prediction and reported mouse phenotypes, suggesting that the mouse reconstruction has similar quality properties as Recon 1.

Initial analysis of sequenced mammalian genomes revealed that the highest number of genes orthologous to Recon 1 genes was found in the mouse while the lowest number was present in the chimpanzee (Table 1). This result was surprising since the sequence homology between human and chimpanzee is ~99% [29] while the sequence homology between human and mouse is in the range of 85-92% [52,53]. This observation reflects the fact that many enzymes involved in human metabolism were discovered by analyzing their mouse orthologues, leading to a high likelihood that mouse metabolic enzymes are known in human and thus present in Recon 1.

The initial search for homologous genes indicated that a functional mouse reconstruction could be produced based on Recon 1. Using two different approaches, we created four versions of a draft mouse model (Figure 1). These draft models were then completed by automated and manual gap filling analysis and validated against 260 FBA-based tests. This effort resulted in iMM1415, which accounts for more genes and reactions, distributed over eight cellular compartments, than previously published reconstructions (Table 2) in addition to its representation of eight cellular compartments. Thus, the mouse metabolic reconstruction presented herein is the most comprehensive reconstruction available to date. However, its usage of the human Recon 1 for reactions information (including cellular location and reversibility) risks that the reactions included are skewed towards human metabolism and that reactions unique to the mouse might be missed.

A surprisingly high number of non-gene-associated reactions are included in Recon 1. Many of those are intra-cellular transport reactions, demand or exchange reactions with sparse literature supporting their existence. They were included to provide a functional model of human metabolism across the eight cellular compartments and were therefore also added to iMM1415. We feel that the gain for a highly functional model by their full inclusion supersedes the risk of artificially inflating the properties of the mouse reconstruction. Furthermore, 30-40% of all known enzymes with an EC number are orphan meaning that their biochemical activity is well characterized while the encoding gene is unknown in any organism [54]. Many of these enzymes (13-37%) are within major metabolism pathways [55] suggesting that metabolic reconstructions of even well-annotated organisms will have a set of non-gene associated reactions in order to ensure their correct functionality. This observation also argues against reconstructing or analyzing metabolic networks based solely on genomic data as they are likely to have numerous missing metabolic functions. Also, the non-gene associated reactions are an extremely interesting area of future research as they suggest pathways and enzymes that require further experimental exploration [55]. Furthermore, several reactions leading to metabolic dead ends in the model were included in Recon 1 and subsequently in our mouse reconstruction. They highlight missing links in knowledge and form testable hypotheses that should be further investigated. Any effort of a consensus metabolic reconstruction of the mouse should focus on reviewing the non-gene-associated reactions and those leading to dead-ends in the model in detail.

Our approach could in theory also be used to construct high quality metabolic reconstructions for other mammals. Given our results (Table 1), it is likely that a rat reconstruction could be obtained using a similar approach as presented here (Figure 1). However, the resulting network is expected to be less complete, limiting its application and requiring more manual curation. Metabolic reconstructions of other mammals will be of insufficient quality and predictive potential using the presented approach due to the low number of homologous genes with Recon1 genes.

The phenotype prediction properties of metabolic models have been extensively applied for microorganisms, however, only limited studies exists for mammals [20,56,57]. The mouse is an ideal model organism for testing and validating phenotypic properties since multiple inbred strains, several thousand gene knockout strains and various cell lines exist [27] including knockouts of many metabolic genes. We found that the majority of predicted essential genes also had a lethal phenotype *in vivo*. Interestingly, the majority of the *in silico *essential genes were found to be within cholesterol metabolism, indicating both the importance and mutation vulnerability of this metabolism pathway. This observation is in concordance with results based on an earlier mouse metabolic model, where the majority of essential genes were from cholesterol metabolism [24]. These results should guide further research and might be helpful in understanding human disease based on lipid abnormalities, such as atherosclerosis [24]. However, many more genes were identified in mouse knockout databases [27] which result in a lethal phenotype *in vivo *but had a non-lethal phenotype *in silico*. The disagreement could result from the non-tissue specificity of iMM1415 or incomplete biomass reaction and highlights starting points for future research to further our insight into mouse metabolism.

The majority of genes for which an *in vivo *experimental knockout strain has been developed have a non-lethal phenotype. Similarly, *in silico *knockout simulations resulted in little or no perturbation of the mouse metabolic network. Recently, it was shown for yeast that even though deletion of ~80% of yeast genes resulted in no apparent phenotype when grown in rich medium, a measurable growth phenotype was observed for ~97% of yeast genes when the medium was either depleted of certain ingredients or biologically active compounds added to the medium [58]. Furthermore, virtually all genes were essential under some growth conditions [58]. It is an important subject of future research to see if the mammalian models suggest similar findings, as such analysis might further understanding of both complex genotypes and the effects of environmental factors on disease pathogenesis.

## Conclusions

Here, we have created a reconstruction of mouse metabolism based on sequence homology, using the highly detailed Recon 1 of human metabolism as a basis for our reconstruction. The model has been rigorously validated using 260 flux balance analysis based tests. The resulting reconstruction, iMM1415, is to date the most comprehensive reconstruction of mouse metabolism. Our phenotype simulation results suggest that the current quality of both the human Recon 1 and the derived iMM1415 models are sufficient for phenotype predictions. The existence of these two reconstructions should encourage the creation of detailed metabolic network reconstructions for other mammals. With the ongoing international knockout mouse project http://www.knockoutmouse.org aiming to produce knockout of all protein coding genes within the mouse genome and the Collaborative Cross project aiming to crossbreed eight inbred strains of mice [59], the validation of mammalian metabolic models should become extensive. Also, the sequencing of more inbred strains of experimental mice will provide new opportunities in studying the effects of genetic variability on metabolism, utilizing the mouse reconstruction as a data analysis platform. The existent and emerging reconstructions should be joined in a collaborative effort to reach a consensus reconstruction of the mouse metabolism to maximize its accuracy and utilization properties. Reconstruction jamboree meetings have been held for various organisms to obtain consensus metabolic reconstructions and to increase their content in a community driven approach [4,60]. Thoroughly validated genome-scale reconstructions should provide a broad platform for studying mammalian metabolism and can form the basis for developing therapeutic interventions related to pathological metabolic states [38,42-44,48,51,61].

## Methods

All model calculations were done in MATLAB version 2009a (The Mathworks Inc.) using the COBRA toolbox [6] and the Mosek linear solver (Mosek ApS, Denmark). Statistical tests and figure preparation were done in R, version 2.9.1 (The R foundation, Austria). In-house scripts for data processing were written in Java and are available upon request from the authors.

### Flux balance analysis and flux variability analysis

Given a reconstructed metabolic network on a mathematical form with appropriate constraints on each reaction (representing reversibility, maximum and minimum flux etc.) and a biological objective Z (such as biomass), flux balance analysis (FBA) calculates a set of network fluxes that maximize Z. It has several derived applications used to further analyze the flow of metabolites through a metabolic model [62]. One such application is flux variability analysis (FVA) [63]. FVA uses linear programming methods to find the minimal and maximal flux values that can be achieved by each reaction while contributing to maximal production of the objective reaction.

### Creation of draft reconstructions

The entire HomoloGene build 52 was downloaded on 11/17/2008 [28]. We extracted data from the dataset for all 1,496 genes in Recon 1 [17]. For each of the 20 species in the HomoloGene database, we then checked for the availability of a homologous gene for each Recon 1 gene. Next we created draft reconstructions for each species using two different approaches as described in the result section. The draft reconstructions were converted into condition-specific draft models [4]. All draft models were checked for functionality by optimizing for biomass while allowing unrestricted uptake of metabolites (v_min, metabolite _≥ -1000 mmol/gdw hr). FVA was performed on the draft models to determine number of reactions with a non-zero flux. Following FVA on each draft model, we drew phylogenetic trees of the results and compared to phylogenetic tree based on all transcripts of protein domain sequence from the SuperFamily database [64].

### Gap filling

Gap filling was done using the SMILEY algorithm, as described elsewhere [7]. We downloaded and used the entire KEGG database (accessed at 9/15/2009) [31] as universal reaction database. Additionally, we created a database of transport and exchange reaction for all metabolites in Recon1 and the KEGG database. These two databases were used to determine candidate reactions to fill gaps in the non-compartmentalized draft model. The gap filling algorithm was set to run 40 iterations. Each solution was then manually checked for applicability (such as the feasibility of the suggested directionality) before choosing a proposed solution.

### Validation

For validation of the mouse reconstruction, we used a modification of the validation process used in the creation of the human Recon 1 network. Each of the 288 validation tests used for Recon 1 was manually checked for applicability in the mouse. This was done by manually reviewing each validation test using Comparative Pathway Analyzer 1.0 [30]. If all enzymes participating in validation test of choice existed both in human and mouse, the test was used in the mouse validation. This resulted in 260 validation tests for the mouse model. The model was then improved manually in an iterative manner by checking the reactions involved in failed tests until it passed all validation tests.

### Simulations

Information on mouse knockout phenotypes were gained from the Mouse Genome Informatics site (http://www.informatics.jax.org, accessed on 1/12/2010) [27]. For each gene whose knockout was simulated, a model with the corresponding reaction bounds set to zero was created. Both the wild type and knockout simulation type models allowed cellular uptake of vital amino acids, vital fatty acids, glucose, oxygen, hydrogen, sulfur oxide, phosphate and ions while optimizing for biomass (detailed exchange constraints listing is in Additional file 7). As computational methods do not allow direct sampling of the flux solution space for large networks[65,66], we performed FVA[63] to estimate the range of flux values for each reaction resulting in the optimal solution. By comparing the FVA for the wild type model and knockout models, each reaction was then assigned a status of decreased flux capacity (i.e., reduced v_max,i_), no change or increased flux capacity. For an unbiased selection of interesting metabolic subsystems, we counted number of reactions with increased and decreased flux ranges and calculated a single value Chi-Square test with 1 degree of freedom comparing the observation against even probability of increased and decreased flux capacity. Since independent tests were done for each of the 10 major subsystems, a *p-value *of 0.05/10 was considered statistically significant to correct for multiple testing.

## Authors' contributions

MIS, NJ, ES, IT and BOP conceived of the study. MIS, NJ and IT created and validated the models. MIS performed phenotype simulations. MIS drafted the manuscript. MIS, ES, IT and BOP revised the manuscript. All authors read and approved the final manuscript.

## Supplementary Material

Additional file 1 Supplemental file S1: Absolute number and percentage of Recon 1 genes found in all species within the HomoloGene database. Click here for file

Additional file 2 Supplemental file S2: Details of KEGG search for unique reactions in mouse and human using Comparative Pathway Analyzer 1.0. Click here for file

Additional file 3 Supplemental file S3: List of 260 flux balance analysis tests used for validation of the mouse reconstruction. Click here for file

Additional file 4 Supplemental file S4: List of genes added again during the validation process and the corresponding metabolic subgroups. Click here for file

Additional file 5 Supplemental file S5: The iMM1415 model of mouse metabolism in a SMBL format. Click here for file

Additional file 6 Supplemental file S6: A detailed reaction list of all reactions in the iMM1415, including the biomass reaction. Click here for file

Additional file 7 Supplemental file S7: Uptake rates for exchange reactions in the mouse reconstruction under minimal medium conditions. Click here for file

Additional file 8 Supplemental file S8: A list of genes predicted to be essential in the mouse metabolic network, along with bibliographic information if available. Click here for file
