# Supplementary material for: A detailed genome-wide reconstruction of mouse metabolism based on human Recon 1
Source: BMC Syst Biol. 2010 Oct 19;4:140. doi: 10.1186/1752-0509-4-140 (PMC2978158; doi:10.1186/1752-0509-4-140)
Supplement: Additional file 1 — Supplemental file S1: Absolute number and percentage of Recon 1 genes found in all species within the HomoloGene database. [file 1752-0509-4-140-S1.PDF]

Absolute number and percentage of Recon 1 genes found in all species within the HomoloGene database.

Species	Number of orthologus genes	Percentage
Mus musculus	1415	97%
Rattus norvegicus	1339	91%
Canis lupus familiaris	1307	89%
Bos taurus	1279	87%
Pan troglodytes	1221	83%
Danio rerio	1200	82%
Gallus gallus	1051	72%
Drosophila melanogaster	662	45%
Anopheles gambiae	635	43%
Caenorhabditis elegans	631	43%
Arabidopsis thaliana	501	34%
Oryza sativa	470	32%
Magnaporthe grisea	364	25%
Neurospora crassa	331	23%
Saccharomyces cerevisiae	287	20%
Kluyveromyces lactis	269	18%
Schizosaccharomyces pombe	253	17%
Eremothecium gossypii	245	17%
Plasmodium falciparum	76	5%
