# Supplementary material for: A detailed genome-wide reconstruction of mouse metabolism based on human Recon 1
Source: BMC Syst Biol. 2010 Oct 19;4:140. doi: 10.1186/1752-0509-4-140 (PMC2978158; doi:10.1186/1752-0509-4-140)
Supplement: Additional file 2 — Supplemental file S2: Details of KEGG search for unique reactions in mouse and human using Comparative Pathway Analyzer 1.0. [file 1752-0509-4-140-S2.PDF]

KEGG map available	Transporters	KEGG map not available	Unique mouse enzymes according to Comparative Pathway Analyzer
Alanine and Aspartate Metabolism	Transport, Endoplasmic Reti	Blood Group Biosynthesis	E.C. Number – note each enzyme can induce several reactions
Alkaloid biosynthesis II	Transport, Endoplasmic Reti	Carnitine shuttle	1.1.1.103
Aminosugar Metabolism	Transport, Extracellular	Chondroitin sulfate degradation	1.1.3.8
Arginine and Proline Metabolism	Transport, Golgi Apparatus	CYP Metabolism	1.11.1.12
Ascorbate and Aldarate Metabolism	Transport, Lysosomal	Heme Biosynthesis	1.14.182
beta-Alanine metabolism	Transport, Mitochondrial	Heme Degradation	1.7.3.3
Bile Acid Biosynthesis	Transport, Nuclear	Heparan sulfate degradation	2.7.7.14
Biotin Metabolism	Transport, Peroxisomal	Hyaluronan Metabolism	6.3.5.6
Butanoate Metabolism		IMP Biosynthesis	6.3.5.7
C5-Branched dibasic acid metabolism		Keratan sulfate biosynthesis	
Cholesterol Metabolism		Keratan sulfate degradation	
Chondroitin / heparan sulfate biosynthesis		Miscellaneous	
Citric Acid Cycle		Nucleic acid degradation	**Unique human enzymes according to Comparative Pathway Analys**
CoA Biosynthesis		Nucleotides	E.C. Number – note each enzyme can induce several reactions
CoA Catabolism		Others	1.1.1.188
Cysteine Metabolism		R Group Synthesis	1.1.1.2
D-alanine metabolism		ROS Detoxification	1.1.1.26
D-arg and D-orn metabolism		Salvage Pathway	1.1.1.29
Eicosanoid Metabolism		Stilbene, coumarine and lignin biosynthesis	1.1.1.50
Fatty acid activation		Tetrahydrobiopterin	1.1.1.53
Fatty acid elongation		Triacylglycerol Synthesis	1.1.1.71
Fatty Acid Metabolism		Vitamin B12 Metabolism	1.1.1.81
Fatty acid oxidation		Vitamin B6 Metabolism	1.14.13.48
Fatty acid oxidation, peroxisome		Vitamin D	1.14.13.49
Folate Metabolism			1.14.13.80
Fructose and Mannose Metabolism			1.2.1.19
Galactose metabolism			1.2.3.1
Glutamate metabolism			1.2.3.8
Glutathione Metabolism			1.5.1.7
Glycerophospholipid Metabolism			2.1.1.4
Glycine, Serine, and Threonine Metabolism			2.1.1.49
Glycolysis/Gluconeogenesis			2.3.1.65
Glycosylphosphatidylinositol (GPI)-anchor biosynthesis			2.4.1.80
Glyoxylate and Dicarboxylate Metabolism			2.4.2.17
Histidine Metabolism			2.4.2.9
Inositol Phosphate Metabolism			2.6.1.22
Limonene and pinene degradation			2.7.1.59
Lysine Metabolism			2.7.1.60
Methionine Metabolism			2.7.4.15
N-Glycan Biosynthesis			2.7.6.2
N-Glycan Degradation			2.7.8.2
Nucleotide Sugar Metabolism			23.2.1.28
O-Glycan Biosynthesis			3.2.1.21
Oxidative Phosphorylation			3.2.1.3
Pentose and Glucuronate Interconversions			4.1.1.45
Pentose Phosphate Pathway			5.1.3.14
Phenylalanine metabolism			5.5.1.4
Propanoate Metabolism			
Purine Catabolism			
Pyrimidine Biosynthesis			
Pyrimidine Catabolism			
Pyruvate Metabolism			
Riboflavin Metabolism			
Selenoamino acid metabolism			
Sphingolipid Metabolism			
Starch and Sucrose Metabolism			
Steroid Metabolism			
Taurine and hypotaurine metabolism			
Thiamine Metabolism			
Tryptophan metabolism			
Tyr, Phe, Trp Biosynthesis			
Tyrosine metabolism			
Ubiquinone Biosynthesis			
Urea cycle/amino group metabolism			
Valine, Leucine, and Isoleucine Metabolism			
Vitamin A Metabolism			
