# Supplementary material for: A detailed genome-wide reconstruction of mouse metabolism based on human Recon 1
Source: BMC Syst Biol. 2010 Oct 19;4:140. doi: 10.1186/1752-0509-4-140 (PMC2978158; doi:10.1186/1752-0509-4-140)
Supplement: Additional file 3 — Supplemental file S3: List of 260 flux balance analysis tests used for validation of the mouse reconstruction. [file 1752-0509-4-140-S3.PDF]

**List of 260 Flux balance analysis tests used for validation of the mouse reconstruction.**

ATP max, aerobic, glc
ATP max, anaerobic, glc
ATP max, aerobic, citrate
ATP max, aerobic, etoh
ATP max, aerobic, glu-L
ATP max, aerobic, gln-L
ATP max, aerobic, gly
ATP max, aerobic, lac-L
ATP max, aerobic, pro-L
gthrd reduces h2o2, GTHP [c]
gthrd reduces h2o2, GTHP [e]
gthrd reduces h2o2, GTHP [m]
gly -> co2 + nh4
Human Recon 1 test mouse biomass
3pg[c] -> gly[c]
3pg[c] -> ser-L[c]
4abut[c] -> succ[m]
4hpro-LT[m] -> glx[m]
5aop[c] -> pheme[c]
aact[c] -> mthgxl[c]
acac[m] -> acetone[m]
acac[m] -> bhb[m]
acald[c] -> ac[c]
accoa[c] -> pmtcoa[c]
pmtcoa[c] -> malcoa[m]
acetone[c] -> mthgxl[c]
acgal[c] -> udpacgal[c]
acorn[c] -> orn[c]
adrnl[c] -> 34dhoxpeg[c]
akg[c] -> glu-L[c] [ALATA_L]
akg[c] -> glu-L[c] [ASPTA]
akg[m] -> oaa[m]
akg[m] -> glu-L[m]
akg[m] -> glu-L[m] [ASPTAm]
ala-B[c] -> msa[m]
ala-D[c] -> pyr[c]
ala-L[c] -> ala-D[c]
ala-L[c] -> pyr[c]
arachd[c] -> malcoa[m]
arachd[r] -> txa2[r]
arg-L[c] -> creat[c]
arg-L -> glu-L [m]
arg-L -> no
arg-L[c] -> pcreat[c]
ascb-L[c] -> eryth[c]
ascb-L[c] -> lyxnt[c]
ascb-L[c] -> thrnt[c]
ascb-L[c] -> xylnt[c]
asn-L[c] -> oaa[c]
asp-L[c] + hco3[c] -> arg-L[c]
asp-L[c] -> ala-B[c]
asp-L[c] -> asn-L[c]
asp-L[c] -> argsuc[c], asp-L -> fum [via argsuc], 1
argsuc[c] -> fum[c], asp-L -> fum [via argsuc], 2
asp-L[c] -> dcamp[c], asp-L -> fum [via dcamp], 1
dcamp[c] -> fum[c], asp-L -> fum [via dcamp], 2
dcamp[c] -> fum[c], asp-L -> fum [via dcamp], 3
asp-L[c] -> oaa[c]
carn -> ala-B
chol[c] + dag_hs[c] -> pe_hs[c]
choline -> betaine [glyb] -> glycine, 1 [m]
choline -> betaine [glyb] -> glycine, 2 [m]
coke[r] -> pecgoncoa[r]

core2[g] -> ksii_core2[g]
core4[g] -> ksii_core4[g]
cspg_a[ly] -> gal[ly] + glcur[ly] + xyl-D[ly]
cspg_b[ly] -> gal[ly] + glcur[ly] + xyl-D[ly]
cspg_c[ly] -> gal[ly] + glcur[ly] + xyl-D[ly]
cspg_d[ly] -> gal[ly] + glcur[ly] + xyl-D[ly]
cspg_e[ly] -> gal[ly] + glcur[ly] + xyl-D[ly]
cys-L + glu-L + gly -> ghtrd
cys-L -> 3sala -> so4, 1
cys-L -> 3sala -> so4, 2
cys-L[c] -> hyptaur[c]
cystine [Lcystin] -> cys-L
dhap[c] -> mthgxl[c]
dmpp[c] -> ggdp[c]
dna[n] -> dna5mtc[n]
dolichol_L[c] -> dolmanp_L[r]
dolichol_L[c] -> g3m8mpdol_L[r]
dolichol_U[c] -> dolmanp_U[r]
dolichol_U[c] -> g3m8mpdol_U[r]
dopa[c] -> homoval[c]
etoh[c] -> acald[c]
f6p[c] + g3p[c] -> r5p[c]
frdp[c] -> dolichol_L[r]
frdp[c] -> dolichol_U[r]
ade[c] -> amp[c]
adn[c] -> urate[x]
adp[c] -> datp[n]
cdp[c] -> dctp[n]
cmp[c] -> cytd[c]
cytd[c] -> ala-B[c]
dcmp[c] -> ala-B[c]
gdp[c] -> dgtp[n]
gln-L + HCO3 -> UMP[c]
gsn[c] -> urate[x]
gua[c] -> gmp[c]
hxan[c] -> imp[c]
imp[c] -> atp[c]
imp[c] -> gtp[c]
imp[c] -> urate[x]
prpp[c] -> imp[c]
pydx[c] -> pydx5p[c]
thmmp[e] -> thmpp[c]
thmmp[e] -> thmpp[m]
tyr-L[m] -> q10[m]
udp[c] -> dttp[n]
ump[c] -> ala-B[c]
fru[c] -> dhap[c]
fru[c] -> g3p[c]
fuc-L[c] -> gdpfuc[c]
fum[m] -> oaa[m]
g1p[c] -> dtdprmn[c]
g3p[c] -> mthgxl[c]
g6p[c] -> r5p[c]
g6p[c] -> ru5p-D[c]
gal[c] -> glc-D[c]
gal[c] -> udpgal[c]
galgluside_hs[g] -> galgalgalthcrm_hs[g]
galgluside_hs[g] -> acgagbside_hs[g]
galgluside_hs[g] -> acnacngalgbside_hs[g]
galgluside_hs[g] -> gd1b2_hs[g]
galgluside_hs[g] -> gd1c_hs[g]
galgluside_hs[g] -> gp1c_hs[g]
galgluside_hs[g] -> gq1balpha_hs[g]
gam6p[c] -> uacgam[c]
gdpmann[c] -> gdpfuc[c]

glc-D[c] -> inost[c]
glc-D[c] -> lac-L[c] + atp[c] + h2o[c]
glc-D[c] -> lac-D[c]
glc-D[c] -> lcts[g]
glc-D[c] -> pyr[c]
gln-L[c] -> nh4[c]
gln-L[m] -> glu-L[m]
gln-L[m] -> glu-L[m]
glu5sa[c] -> pro-L[c]
glu-L[c] -> 4abut[c]
glu-L[c] -> gln-L[c]
glu-L -> pro-L
glu-L[m] -> akg[m]
gluside_hs[g] -> galgluside_hs[g]
glx[m] -> glyclt[m]
gly[c] -> ser-L[c] -> pyr[c], 1
gly[c] -> ser-L[c] -> pyr[c], 2
glyc[c] -> glc-D[c]
glyc[c] + Rtotal[c] + Rtotal2[c] -> dag_hs[c]
glyc[c] + Rtotal[c] -> tag_hs[c]
glyclt[c] -> gly[c]
glygn2[c] -> glc-D[c]
glygn2[e] -> glc-D[e]
glx[c] -> oxa[c]
ha[l] -> acgam[l] + glcur[l]
his-L[c] -> glu-L[c]
his-L[c] -> hista[c]
hista[c] -> 3mlda[c]
hista[c] -> im4ac[c]
hmgcoa[x] -> chsterol[r]
hmgcoa[x] -> frdp[x]
hmgcoa[x] -> xoldiolone[r]
hpyr[c] -> 2pg[c]
hpyr[c] -> glyclt[c]
hpyr[c] -> glyc-S[c]
hspg[ly] -> gal[ly] + glcur[ly] + xyl-D[ly]
hyptaur[c] -> taur[x]
ile-L[c] -> accoa[c]
inost[c] -> pail_hs[c]
inost[c] -> pail45p_hs[c]
inost[c] -> pail4p_hs[c]
inost[c] -> xu5p-D[c]
ipdp[x] -> sql[r]
itacon[m] -> pyr[m]
ksi[l] -> man[l] + acgam[l]
ksii_core2[l] -> Ser/Thr[l]
ksii_core4[l] -> Ser/Thr[l]
l2fn2m2masn[g] -> ksi[g]
lac-L[c] -> glc-D[c]
Lcyst[c] -> taur[x]
leu-L[c] -> accoa[c]
lys-L[c] -> accoa[m] [via saccrp-L pathway]
lys-L[x] -> aacoa[m] [via Lpipecol pathway]
m8masn[r] -> nm4masn[g]
man[c] -> gdpmann[c]
man6p[c] -> kdn[c]
mescon[m] -> pyr[m]
met-L[c] -> cys-L[c]
mi145p[c] -> inost[c]
msa[c] -> ala-B[c]
mthgxl[c] -> 12ppd-S[c]
mthgxl[c] -> lac-D[c]
n2m2nmasn[l] -> man[l] + acgam[l]
nm4masn[g] -> l2fn2m2masn[g]
nm4masn[g] -> n2m2nmasn[g]

nm4masn[g] -> s2l2fn2m2masn[g]
o2- -> h2o2 -> o2 + h2o, 1
o2- -> h2o2 -> o2 + h2o, 2
orn[c] -> nh4[c]
orn[c] -> ptrc[c]
pail45p[c] -> mi145p[c]
phe-L[c] -> pac[c]
phe-L[c] -> pacald[c]
phe-L[c] -> peamn[c]
phe-L[c] -> phaccoa[c]
phe-L[c] -> pheacgln[c]
phe-L[c] -> phpyr[c]
phe-L[c] -> tyr-L[c]
pheme[c] -> bilirub[c]
pmtcoa[c] -> crmp_hs[c]
pmtcoa[c] -> sphmyln_hs[c]
ppcoa[m] -> succoa[m]
pro-L[c] -> glu-L[c]
pyr -> fad[m] + h[m]
pyr[c] -> lac-D[c]
pyr -> nad[m] + h[m]
pyr[c] -> accoa[m] + co2[c] + nadh[m]
pyr[c] -> ala-L[c], 1
pyr[c] -> ala-L[c], 2
s2l2fn2m2masn[l] -> man[l] + acgam[l]
selmeth[c] -> selnp[c]
Ser/Thr[g] + udpacgal[g] -> core2[g]
Ser/Thr[g] + udpacgal[g] -> core4[g]
Ser/Thr[g] + udpacgal[g] -> Tn_antigen[g]
Ser/Thr[g] + udpacgal[g] -> sTn_antigen[g]
Ser-Gly/Ala-X-Gly[r] -> cs_pre[g]
Ser-Gly/Ala-X-Gly[r] -> cspg_a[g]
Ser-Gly/Ala-X-Gly[r] -> cspg_c[g]
Ser-Gly/Ala-X-Gly[r] -> cspg_d[g]
Ser-Gly/Ala-X-Gly[r] -> cspg_e[g]
Ser-Gly/Ala-X-Gly[r] -> hspg[g]
Ser-Gly/Ala-X-Gly[r] -> cspg_b[g]
ser-L[c] -> cys-L[c]
so4[c] -> paps[c]
srtn[c] -> f5hoxkyn[c]
strch1[e] -> glc-D[e]
succoa[m] -> oaa[m]
trp-L[c] -> ppcoa[c]
trp-L[c] -> anth[c]
trp-L[c] -> id3acald[c]
trp-L[c] -> kynate[c]
trp-L[c] -> Lfmkynr[c]
trp-L[c] -> Lkynr[c]
trp-L[c] -> nformanth[c]
trp-L[c] -> quln[c]
trp-L[c] -> srtn[c]
Tyr-ggn[c] -> glygn2[c]
tyr-L[c] -> 34hpp[c]
tyr-L[c] -> 4hphac[c]
tyr-L[c] -> adrnl[c]
tyr-L[c] -> dopa[c]
tyr-L[c] -> fum[c] + acac[c]
tyr-L[c] -> melanin[c]
tyr-L[c] -> nrpphr[c]
uacgamv[c] + udpglcur[c] -> ha[e]
uacgam[c] -> m8masn[r]
udpglcur[c] -> xu5p-D[c]
ura[c] -> ala-B[c]
val-L[c] -> 3aib[c]
val-L[c] -> succoa[m]

xoltriol[m] -> thcholstoic[m]
xylu-D[c] -> glyclt[c]
