# Supplementary material for: A detailed genome-wide reconstruction of mouse metabolism based on human Recon 1
Source: BMC Syst Biol. 2010 Oct 19;4:140. doi: 10.1186/1752-0509-4-140 (PMC2978158; doi:10.1186/1752-0509-4-140)
Supplement: Additional file 4 — Supplemental file S4: List of genes added again during the validation process and the corresponding metabolic subgroups. [file 1752-0509-4-140-S4.PDF]

List of reactions added again during the validation process and the corresponding metabolic subgroups.

Gene Name	Metabolism subgroup
'IPDDIx'	'Cholesterol Metabolism'
'GLCAT2g'	'Chondroitin / heparan sulfate biosynthesis'
'GLCAT3g'	'Chondroitin / heparan sulfate biosynthesis'
'GLCAT4g'	'Chondroitin / heparan sulfate biosynthesis'
'GLCAT5g'	'Chondroitin / heparan sulfate biosynthesis'
'PSDm_hs'	'Glycerophospholipid Metabolism'
'HPYRR2x'	'Glyoxylate and Dicarboxylate Metabolism'
'MI1345PP'	'Inositol Phosphate Metabolism'
'MI145PP'	'Inositol Phosphate Metabolism'
'PI45PLC'	'Inositol Phosphate Metabolism'
'S23T3g'	'Keratan sulfate biosynthesis'
'S6T10g'	'Keratan sulfate biosynthesis'
'S6T11g'	'Keratan sulfate biosynthesis'
'S6T12g'	'Keratan sulfate biosynthesis'
'S6T13g'	'Keratan sulfate biosynthesis'
'S6T14g'	'Keratan sulfate biosynthesis'
'S6T16g'	'Keratan sulfate biosynthesis'
'S6T17g'	'Keratan sulfate biosynthesis'
'S6T1g'	'Keratan sulfate biosynthesis'
'S6T2g'	'Keratan sulfate biosynthesis'
'S6T4g'	'Keratan sulfate biosynthesis'
'S6T5g'	'Keratan sulfate biosynthesis'
'S6T6g'	'Keratan sulfate biosynthesis'
'S6T7g'	'Keratan sulfate biosynthesis'
'S6T8g'	'Keratan sulfate biosynthesis'
'S6T9g'	'Keratan sulfate biosynthesis'
'DOLPMT3_	'N-Glycan Biosynthesis'
'DOLPMT3_	'N-Glycan Biosynthesis'
'M1316Mg'	'N-Glycan Biosynthesis'
'ATPS4m'	'Oxidative Phosphorylation'
'NADH2-u1(	'Oxidative Phosphorylation'
'AMY1e'	'Starch and Sucrose Metabolism'
'AMY2e'	'Starch and Sucrose Metabolism'
'GGNG'	'Starch and Sucrose Metabolism'
'GLGNS1'	'Starch and Sucrose Metabolism'
