# Supplementary material for: A detailed genome-wide reconstruction of mouse metabolism based on human Recon 1
Source: BMC Syst Biol. 2010 Oct 19;4:140. doi: 10.1186/1752-0509-4-140 (PMC2978158; doi:10.1186/1752-0509-4-140)
Supplement: Additional file 7 — Supplemental file S7: Uptake rates for exchange reactions in the mouse reconstruction under minimal medium conditions. [file 1752-0509-4-140-S7.PDF]

Name	Lower bounda mmol/gdw/hr	Upper bounda mmol/gdw/hr
'DM_13-cis-oretn(n)'	0	0
'DM_13-cis-retn(n)'	0	0
'DM_Asn-X-Ser/Thr(ly)'	0	100000
'DM_Ser-Gly/Ala-X-Gly(ly)'	0	100000
'DM_Ser/Thr(ly)'	0	100000
'DM_atp(c)'	0	100000
'DM_avite1(c)'	0	100000
'DM_avite2(c)'	0	100000
'DM_bvite(c)'	0	100000
'DM_core5(g)'	0	100000
'DM_core7(g)'	0	100000
'DM_core8(g)'	0	100000
'DM_datp(m)'	0	100000
'DM_datp(n)'	0	100000
'DM_dctp(m)'	0	100000
'DM_dctp(n)'	0	100000
'DM_dem2emgacpail_prot_hs(r)'	0	100000
'DM_dgpi_prot_hs(r)'	0	100000
'DM_dgtp(m)'	0	100000
'DM_dgtp(n)'	0	100000
'DM_dsT_antigen(g)'	0	100000
'DM_dttp(m)'	0	100000
'DM_dttp(n)'	0	100000
'DM_ethamp(r)'	0	100000
'DM_gncore2(g)'	0	100000
'DM_gpi_sig(er)'	0	100000
'DM_hretn(n)'	0	100000
'DM_kdn(c)'	0	100000
'DM_m(em)3gacpail_prot_hs(r)'	0	100000
'DM_melanin(c)'	0	100000
'DM_mem2emgacpail_prot_hs(r)'	0	100000
'DM_n5m2masn(g)'	0	100000
'DM_oretn(n)'	0	100000
'DM_sTn_antigen(g)'	0	100000
'DM_sprm(c)'	0	100000
'DM_yvite(c)'	0	100000
'DM_T_antigen(g)'	-1	100000
'EX_10fthf(e)'	0	100000
'EX_10fthf5glu(e)'	0	100000
'EX_10fthf6glu(e)'	0	100000
'EX_10fthf7glu(e)'	0	100000
'EX_11-cis-retfa(e)'	0	100000
'EX_13-cis-retnglc(e)'	0	100000
'EX_1glyc_hs(e)'	0	100000
'EX_1mncam(e)'	0	100000
'EX_2425dhvitd2(e)'	0	100000
'EX_2425dhvitd3(e)'	0	100000

'EX_24nph(e)'	0	100000
'EX_25hvitd2(e)'	0	100000
'EX_25hvitd3(e)'	0	100000
'EX_2hb(e)'	0	100000
'EX_2mcit(e)'	0	100000
'EX_34dhoxpeg(e)'	0	100000
'EX_34dhphe(e)'	0	100000
'EX_35cgmp(e)'	0	100000
'EX_3aib(e)'	0	100000
'EX_3aib-D(e)'	0	100000
'EX_3mlda(e)'	0	100000
'EX_4abut(e)'	0	100000
'EX_4hdebrisoquine(e)'	0	100000
'EX_4hphac(e)'	0	100000
'EX_4mptnl(e)'	0	100000
'EX_4mtolbutamide(e)'	0	100000
'EX_4nph(e)'	0	100000
'EX_4nphsf(e)'	0	100000
'EX_4pyrdx(e)'	0	100000
'EX_5adtststerone(e)'	0	100000
'EX_5adtststeroneglc(e)'	0	100000
'EX_5adtststerones(e)'	0	100000
'EX_5dhf(e)'	0	100000
'EX_5fthf(e)'	0	100000
'EX_5homeprazole(e)'	0	100000
'EX_5htrp(e)'	0	100000
'EX_5mthf(e)'	0	100000
'EX_5thf(e)'	0	100000
'EX_6dhf(e)'	0	100000
'EX_6htststerone(e)'	0	100000
'EX_6thf(e)'	0	100000
'EX_7dhf(e)'	0	100000
'EX_7thf(e)'	0	100000
'EX_9-cis-retfa(e)'	0	100000
'EX_CLPND(e)'	0	100000
'EX_Lcystin(e)'	0	100000
'EX_Rtotal(e)'	0	100000
'EX_Rtotal2(e)'	0	100000
'EX_Rtotal3(e)'	0	100000
'EX_Tyr-ggn(e)'	0	100000
'EX_abt(e)'	0	100000
'EX_ac(e)'	0	100000
'EX_acac(e)'	0	100000
'EX_acald(e)'	0	100000
'EX_acetone(e)'	0	100000
'EX_acgalfucgalacgalfuc12gal14acglcgalgluside_hs(e)'	0	100000
'EX_acgalfucgalacgalfucgalacglcgal14acglcgalgluside_hs(e)'	0	100000
'EX_acgam(e)'	0	100000
'EX_ach(e)'	0	100000

'EX_acn13acngalgbside_hs(e)'	0	100000
'EX_acn23acngalgbside_hs(e)'	0	100000
'EX_acnacngal14acglcgalgluside_hs(e)'	0	100000
'EX_acnacngalgbside_hs(e)'	0	100000
'EX_acngalacglcgal14acglcgalgluside_hs(e)'	0	100000
'EX_ade(e)'	0	100000
'EX_adn(e)'	0	100000
'EX_adp'	0	100000
'EX_adprbp(e)'	0	100000
'EX_adprib(e)'	0	100000
'EX_adrn(e)'	0	100000
'EX_adrnl(e)'	0	100000
'EX_aflatoxin(e)'	0	100000
'EX_ahandrostanglc(e)'	0	100000
'EX_ak2lgchol_hs(e)'	0	100000
'EX_akg(e)'	0	100000
'EX_ala-B(e)'	0	100000
'EX_ala-D(e)'	0	100000
'EX_ala-L(e)'	0	100000
'EX_aldstrn(e)'	0	100000
'EX_amp(e)'	0	100000
'EX_andrstrn(e)'	0	100000
'EX_andrstrnglc(e)'	0	100000
'EX_antipyrene(e)'	0	100000
'EX_apnnox(e)'	0	100000
'EX_appnn(e)'	0	100000
'EX_aprgstrn(e)'	0	100000
'EX_aqcobal(e)'	0	100000
'EX_arab-L(e)'	0	100000
'EX_arach(e)'	0	100000
'EX_arachd(e)'	0	100000
'EX_arg-L(e)'	-1	100000
'EX_ascb-L(e)'	0	100000
'EX_asn-L(e)'	0	100000
'EX_asp-D(e)'	0	100000
'EX_asp-L(e)'	0	100000
'EX_atp(e)'	0	100000
'EX_avite1(e)'	0	100000
'EX_avite2(e)'	0	100000
'EX_bhb(e)'	0	100000
'EX_bildglcur(e)'	0	100000
'EX_bilglcur(e)'	0	100000
'EX_bilirub(e)'	0	100000
'EX_biocyt(e)'	0	100000
'EX_btn(e)'	0	100000
'EX_but(e)'	0	100000
'EX_bvite(e)'	0	100000
'EX_bz(e)'	0	100000
'EX_ca2(e)'	-1	100000

'EX_camp(e)'	0	100000
'EX_caro(e)'	0	100000
'EX_carveol(e)'	0	100000
'EX_cca_d3(e)'	0	100000
'EX_cgly(e)'	0	100000
'EX_chol(e)'	0	100000
'EX_cholate(e)'	0	100000
'EX_chsterol(e)'	0	100000
'EX_chtn(e)'	0	100000
'EX_cit(e)'	0	100000
'EX_cl(e)'	-1	100000
'EX_cmp(e)'	0	100000
'EX_co(e)'	-1	100000
'EX_co2(e)'	-100	100000
'EX_coumarin(e)'	0	100000
'EX_creat(e)'	0	100000
'EX_crmp_hs(e)'	0	100000
'EX_crn(e)'	0	100000
'EX_crtsl(e)'	0	100000
'EX_crtstrn(e)'	0	100000
'EX_crvnc(e)'	0	100000
'EX_csn(e)'	0	100000
'EX_cspg_a(e)'	0	100000
'EX_cspg_b(e)'	0	100000
'EX_cspg_c(e)'	0	100000
'EX_cspg_d(e)'	0	100000
'EX_cspg_e(e)'	0	100000
'EX_cyan(e)'	0	100000
'EX_cys-L(e)'	0	100000
'EX_cytd(e)'	0	100000
'EX_dad-2(e)'	0	100000
'EX_dad-5(e)'	0	100000
'EX_dag_hs(e)'	0	100000
'EX_dcsptn1(e)'	0	100000
'EX_dcyt(e)'	0	100000
'EX_debrisoquine(e)'	0	100000
'EX_dgchol(e)'	0	100000
'EX_dgsn(e)'	0	100000
'EX_dhdascb(e)'	0	100000
'EX_dheas(e)'	0	100000
'EX_dhf(e)'	0	100000
'EX_digalsgalside_hs(e)'	0	100000
'EX_din(e)'	0	100000
'EX_dlnlcg(e)'	0	100000
'EX_dmantipyrine(e)'	0	100000
'EX_dmhptcrn(e)'	0	100000
'EX_dopa(e)'	0	100000
'EX_dopasf(e)'	0	100000
'EX_drib(e)'	0	100000

'EX_duri(e)'	0	100000
'EX_eaflatoxin(e)'	0	100000
'EX_ebastine(e)'	0	100000
'EX_ebastineoh(e)'	0	100000
'EX_eicostet(e)'	0	100000
'EX_elaid(e)'	0	100000
'EX_estradiol(e)'	0	100000
'EX_estradiolglc(e)'	0	100000
'EX_estriolglc(e)'	0	100000
'EX_estroneglc(e)'	0	100000
'EX_estrones(e)'	0	100000
'EX_etoh(e)'	0	100000
'EX_fe2(e)'	-1	100000
'EX_fe3(e)'	-1	100000
'EX_fol(e)'	0	100000
'EX_for(e)'	0	100000
'EX_fru(e)'	0	100000
'EX_fuc-L(e)'	0	100000
'EX_fuc13galacglcgal14acglcgalgluside_hs(e)'	0	100000
'EX_fuc14galacglcgalgluside_hs(e)'	0	100000
'EX_fucacgalfucgalacglcgalgluside_hs(e)'	0	100000
'EX_fucacngal14acglcgalgluside_hs(e)'	0	100000
'EX_fucacngalacglcgalgluside_hs(e)'	0	100000
'EX_fucfuc12gal14acglcgalgluside_hs(e)'	0	100000
'EX_fucfuc132galacglcgal14acglcgalgluside_hs(e)'	0	100000
'EX_fucfucfucgalacglc13galacglcgal14acglcgalgluside_hs(e)'	0	100000
'EX_fucfucfucgalacglcgal14acglcgalgluside_hs(e)'	0	100000
'EX_fucfucgalacglcgalgluside_hs(e)'	0	100000
'EX_fucgal14acglcgalgluside_hs(e)'	0	100000
'EX_fucgalfucgalacglcgalgluside_hs(e)'	0	100000
'EX_fucgalgbside_hs(e)'	0	100000
'EX_gal(e)'	0	100000
'EX_galacglcgalgbside_hs(e)'	0	100000
'EX_galfuc12gal14acglcgalgluside_hs(e)'	0	100000
'EX_galfucgalacglcgal14acglcgalgluside_hs(e)'	0	100000
'EX_galgalfucfucgalacglcgalacglcgal14acglcgalgluside_hs(e)'	0	100000
'EX_galgalgalthcrm_hs(e)'	0	100000
'EX_gam(e)'	0	100000
'EX_gbside_hs(e)'	0	100000
'EX_gchola(e)'	0	100000
'EX_gd1b2_hs(e)'	0	100000
'EX_gd1c_hs(e)'	0	100000
'EX_gdchola(e)'	0	100000
'EX_gdp(e)'	0	100000
'EX_glc(e)'	-1	100000
'EX_gln-L(e)'	0	100000
'EX_glu-L(e)'	0	100000
'EX_gluala(e)'	0	100000
'EX_gly(e)'	0	100000

'EX_glyb(e)'	0	100000
'EX_glyc(e)'	-1	100000
'EX_glyc-S(e)'	0	100000
'EX_glygn2(e)'	0	100000
'EX_glygn4(e)'	0	100000
'EX_glygn5(e)'	0	100000
'EX_gmp(e)'	0	100000
'EX_gp1c_hs(e)'	0	100000
'EX_gp1calpha_hs(e)'	0	100000
'EX_gq1b_hs(e)'	0	100000
'EX_gq1balpha_hs(e)'	0	100000
'EX_gsn(e)'	0	100000
'EX_gt1a_hs(e)'	0	100000
'EX_gthox(e)'	0	100000
'EX_gthrd(e)'	0	100000
'EX_gtp(e)'	0	100000
'EX_gua(e)'	0	100000
'EX_h(e)'	-100	100000
'EX_h2o(e)'	-100	100000
'EX_h2o2(e)'	0	100000
'EX_ha(e)'	0	100000
'EX_ha_pre1(e)'	0	100000
'EX_hco3(e)'	-100	100000
'EX_hcoumarin(e)'	0	100000
'EX_hdca(e)'	-1	100000
'EX_hdcea(e)'	0	100000
'EX_hestratriol(e)'	0	100000
'EX_hexc(e)'	0	100000
'EX_his-L(e)'	-1	100000
'EX_hista(e)'	0	100000
'EX_hom-L(e)'	0	100000
'EX_hpdca(e)'	0	100000
'EX_hspg(e)'	0	100000
'EX_htaxol(e)'	0	100000
'EX_hxan(e)'	0	100000
'EX_i(e)'	-1	100000
'EX_idp(e)'	0	100000
'EX_ile-L(e)'	-1	100000
'EX_imp(e)'	0	100000
'EX_inost(e)'	0	100000
'EX_ins(e)'	0	100000
'EX_k(e)'	-1	100000
'EX_ksi(e)'	0	100000
'EX_ksi_deg1(e)'	0	100000
'EX_ksii_core2(e)'	0	100000
'EX_ksii_core4(e)'	0	100000
'EX_lac-D(e)'	0	100000
'EX_lac-L(e)'	0	100000
'EX_lcts(e)'	0	100000

'EX_leu-L(e)'	-1	100000
'EX_leuktrA4(e)'	0	100000
'EX_leuktrB4(e)'	0	100000
'EX_leuktrC4(e)'	0	100000
'EX_leuktrD4(e)'	0	100000
'EX_leuktrE4(e)'	0	100000
'EX_leuktrF4(e)'	0	100000
'EX_lgnc(e)'	0	100000
'EX_limnen(e)'	0	100000
'EX_lipoate(e)'	0	100000
'EX_lneldc(e)'	0	100000
'EX_lnlc(e)'	-1	100000
'EX_lnlc(e)'	-1	0
'EX_lnlnca(e)'	0	100000
'EX_lnlncg(e)'	0	100000
'EX_lpchol_hs(e)'	0	100000
'EX_lys-L(e)'	-1	100000
'EX_mag_hs(e)'	0	100000
'EX_malt(e)'	0	100000
'EX_malttr(e)'	0	100000
'EX_man(e)'	0	100000
'EX_meoh(e)'	0	100000
'EX_mepi(e)'	0	100000
'EX_mercplaccys(e)'	0	100000
'EX_met-L(e)'	-1	100000
'EX_mthgxl(e)'	0	100000
'EX_n2m2nmasn(e)'	0	100000
'EX_na1(e)'	-1	100000
'EX_nac(e)'	0	100000
'EX_nad(e)'	0	100000
'EX_nadp(e)'	0	100000
'EX_ncam(e)'	0	100000
'EX_nh4(e)'	-100	100000
'EX_nifedipine(e)'	0	100000
'EX_no(e)'	0	100000
'EX_npthl(e)'	0	100000
'EX_nrpphr(e)'	0	100000
'EX_nrpphrsf(e)'	0	100000
'EX_nrvnc(e)'	0	100000
'EX_o2(e)'	-100	100000
'EX_o2s(e)'	0	100000
'EX_oagd3_hs(e)'	0	100000
'EX_oagt3_hs(e)'	0	100000
'EX_ocdca(e)'	0	100000
'EX_ocdcea(e)'	0	100000
'EX_octa(e)'	0	100000
'EX_oh1'	0	100000
'EX_omeprazole(e)'	0	100000
'EX_onpthl(e)'	0	100000

'EX_orn(e)'	0	100000
'EX_oxa(e)'	0	100000
'EX_paf_hs(e)'	0	100000
'EX_pchol_hs(e)'	0	100000
'EX_pe_hs(e)'	0	100000
'EX_peplys(e)'	0	100000
'EX_perillyl(e)'	0	100000
'EX_pglyc_hs(e)'	0	100000
'EX_phe-L(e)'	-1	100000
'EX_pheacgln(e)'	0	100000
'EX_pheme(e)'	0	100000
'EX_phyQ(e)'	0	100000
'EX_phyt(e)'	0	100000
'EX_pi(e)'	-100	100000
'EX_pnto-R(e)'	0	100000
'EX_ppa(e)'	0	100000
'EX_prgstrn(e)'	0	100000
'EX_pro-D(e)'	0	100000
'EX_pro-L(e)'	0	100000
'EX_prostgd2(e)'	0	100000
'EX_prostge1(e)'	0	100000
'EX_prostge2(e)'	0	100000
'EX_prostgf2(e)'	0	100000
'EX_ps_hs(e)'	0	100000
'EX_ptdca(e)'	0	100000
'EX_pydam(e)'	0	100000
'EX_pydx(e)'	0	100000
'EX_pydxn(e)'	0	100000
'EX_pyr(e)'	0	100000
'EX_rbt(e)'	0	100000
'EX_retfa(e)'	0	100000
'EX_retinol(e)'	0	100000
'EX_retinol-9-cis(e)'	0	100000
'EX_retinol-cis-11(e)'	0	100000
'EX_retn(e)'	0	100000
'EX_retnglc(e)'	0	100000
'EX_retpalm'	0	0
'EX_retpalm(e)'	0	0
'EX_rib-D(e)'	0	100000
'EX_ribflv(e)'	0	100000
'EX_s2l2fn2m2masn(e)'	0	100000
'EX_s2l2n2m2masn(e)'	0	100000
'EX_sarcs(e)'	0	100000
'EX_sel(e)'	-1	100000
'EX_ser-D(e)'	0	100000
'EX_ser-L(e)'	0	100000
'EX_sl-L(e)'	0	100000
'EX_so4(e)'	-100	100000
'EX_spc_hs(e)'	0	100000

'EX_sph1p(e)'	0	100000
'EX_sphs1p(e)'	0	100000
'EX_srtn(e)'	0	100000
'EX_strch1(e)'	0	100000
'EX_strch2(e)'	0	100000
'EX_strdnc(e)'	0	100000
'EX_succ(e)'	0	100000
'EX_sucr(e)'	0	100000
'EX_tag_hs(e)'	0	100000
'EX_tagat-D(e)'	0	100000
'EX_taur(e)'	0	100000
'EX_taxol(e)'	0	100000
'EX_tchola(e)'	0	100000
'EX_tcynt(e)'	0	100000
'EX_tdchola(e)'	0	100000
'EX_tethex3(e)'	0	100000
'EX_tetpent3(e)'	0	100000
'EX_tetpent6(e)'	0	100000
'EX_tettet6(e)'	0	100000
'EX_thf(e)'	0	100000
'EX_thm(e)'	0	100000
'EX_thmmp(e)'	0	100000
'EX_thmtp(e)'	0	100000
'EX_thr-L(e)'	-1	100000
'EX_thym(e)'	0	100000
'EX_thymd(e)'	0	100000
'EX_thyox-L(e)'	0	100000
'EX_tmndnc(e)'	0	100000
'EX_tolbutamide(e)'	0	100000
'EX_tre(e)'	0	100000
'EX_triodthy(e)'	0	100000
'EX_triodthysuf(e)'	0	100000
'EX_trp-L(e)'	-1	100000
'EX_tststerone(e)'	0	100000
'EX_tststeroneglc(e)'	0	100000
'EX_tststerones(e)'	0	100000
'EX_tsul(e)'	0	100000
'EX_ttdca(e)'	0	100000
'EX_txa2(e)'	0	100000
'EX_tymsf(e)'	0	100000
'EX_tyr-L(e)'	0	100000
'EX_udp(e)'	0	100000
'EX_ump(e)'	0	100000
'EX_ura(e)'	0	100000
'EX_urate(e)'	0	100000
'EX_urea(e)'	0	100000
'EX_uri(e)'	0	100000
'EX_utp(e)'	0	100000
'EX_vacc(e)'	0	100000

'EX_val-L(e)'	-1	100000
'EX_vitd2(e)'	0	100000
'EX_vitd3(e)'	0	100000
'EX_whddca(e)'	0	100000
'EX_whhdca(e)'	0	100000
'EX_whtststerone(e)'	0	100000
'EX_whttdca(e)'	0	100000
'EX_xolest2_hs(e)'	0	100000
'EX_xolest_hs(e)'	0	100000
'EX_xoltri24(e)'	0	100000
'EX_xoltri25(e)'	0	100000
'EX_xoltri27(e)'	0	100000
'EX_xyl-D(e)'	0	100000
'EX_xylt(e)'	0	100000
'EX_yvite(e)'	0	100000
'sink_citr(c)'	0	0
'sink_pre_prot(er)'	-1	100000
