# Supplementary material for: A detailed genome-wide reconstruction of mouse metabolism based on human Recon 1
Source: BMC Syst Biol. 2010 Oct 19;4:140. doi: 10.1186/1752-0509-4-140 (PMC2978158; doi:10.1186/1752-0509-4-140)
Supplement: Additional file 8 — Supplemental file S8: A list of genes predicted to be essential in the mouse metabolic network, along with bibliographic information if available. [file 1752-0509-4-140-S8.PDF]

Gene Name	**Reactions**	**Subgroup**	**Mutation lethal**	**Comment**	**Reference PMID**
EBP	3-beta-hydroxysteroid-delta(8),delta(7)-isomerase	Cholesterol Metabolism	yes	X-linked genes, homozygous males are non-viable (prenatal lethality)	11309666
DHCR7	7-dehydrocholesterol reductase	Cholesterol Metabolism	yes	Prenatal lethality of homozygotes	15862627
DHCR24	24-dehydrocholesterol reductase	Cholesterol Metabolism	yes	Prenatal lethality of homozygotes	16410790
FDFT1	Squalene synthase	Cholesterol Metabolism	yes	Prenatal lethality of homozygotes	10521476
HSD17B4	C-3 sterol keto reductase, Beta oxidation of long chain fatty acid, 3-hydroxyacyl-CoA dehydrogenase, hydroxysteroid (17-beta) dehydrogenase 4, peroxisomal lumped long chain fatty acid oxidation	Cholesterol Metabolism	yes	Pre/Peri/Postnathal lethality of homozygotes	16766224
NSDHL	C-3 sterol dehydrogenase, C-4 methyl sterol oxidase	Cholesterol Metabolism	yes	X-linked gene, males and homozygous females are non-viable (prenatal lethality)	19631568
SC5DL	Lathosterol oxidase	Cholesterol Metabolism	yes	Perinatal lethality of homozygotes	12812989
SPTLC1	serine palmitoyltransferase, long chain base subunit 1	Sphingolipid Metabolism	yes	Embryonic lethality of homozygotes	16216550
DHFR	dihydrofolate reductase, folate reductase	Folate Metabolism	yes	Embryonic lethality of homozygotes	12024029
PISD	phosphatidylserine decarboxylase, mitochondrial	Glycerophospholipid Metabolism	yes	Embryonic lethality of homozygotes	16192276

PHGDH	phosphoglycerate dehydrogenase	Glycine, Serine, and Threonine Metabolism	yes	Embryonic lethality of homozygotes	14645240
HMGCR	Hydroxymethylglutaryl CoA reductase (ir)	Cholesterol Metabolism	yes	Embryonic lethality of homozygotes	12920113
CBS	cystathionine beta-synthase, selanocystathionine beta-synthase	Methionine Metabolism	yes	Homozygous mice die within 5 weeks after birth	7878023
SPTLC2	serine C-palmitoyltransferase	Sphingolipid Metabolism	yes	Embryonic lethality of homozygotes	16216550
CYP51A1	Cytochrome P450 lanosterol 14-alpha-demethylase	Cholesterol Metabolism	not known		
FDPS	Dimethylallyltranstransferase, geranyltranstransferase	Cholesterol Metabolism	not known		
TMEM23	Sphingomyelin synthase	Sphingolipid Metabolism	not known		
GUK1	Deoxyguanylate kinase, guanylate kinase	Nucleotides	not known		
LSS	Lanosterol synthase	Cholesterol Metabolism	not known		
C20orf155	Cardiolipin synthase	Glycerophospholipid Metabolism	not known		
SC4MOL	C-4 methyl sterol oxidase	Cholesterol Metabolism	not known		
SQLE	Squalene epoxidase, endoplasmic reticular	Cholesterol Metabolism	not known		

PGS1	Phosphatidyl-CMP: glycerophosphate phosphatidyltransferase	Glycerophospholipid Metabolism	not known
PAICS	phosphoribosylaminoimidazole carboxylase, phosphoribosylaminoimidazolesuccinocarboxamide synthase	IMP Biosynthesis	not known
PMVK	phosphomevalonate kinase	Cholesterol Metabolism	not known
UQCR	ubiquinol-6 cytochrome c reductase, Complex III	Oxidative Phosphorylation	not known
CYC1	ubiquinol-6 cytochrome c reductase, Complex III	Oxidative Phosphorylation	not known
DHODH	dihydoorotic acid dehydrogenase (quinone10)	Pyrimidine Biosynthesis	not known
RPIA	ribose-5-phosphate isomerase	Pentose Phosphate Pathway	not known
FVT1	3-Dehydrosphinganine reductase	Sphingolipid Metabolism	not known
QP-C	ubiquinol-6 cytochrome c reductase, Complex III	Oxidative Phosphorylation	not known
MTCYB	ubiquinol-6 cytochrome c reductase, Complex III	Oxidative Phosphorylation	not known
MVD	diphosphomevalonate decarboxylase	Cholesterol Metabolism	not known
MVK	mevalonate kinase (atp)	Cholesterol Metabolism	not known
ATIC	phosphoribosylaminoimidazolecarboxamide formyltransferase, IMP cyclohydrolase	IMP Biosynthesis	not known

ISYNA1	myo-Inositol-1-phosphate synthase	Inositol Phosphate Metabolism	not known
CMPK	cytidylate kinase, UMP kinae	Nucleotides	not known
PFAS	phosphoribosylformylglycinamidine synthase	IMP Biosynthesis	not known
PPAT	glutamine phosphoribosyldiphosphate amidotransferase	IMP Biosynthesis	not known
RRM2	ribonucleoside-diphosphate reductase	Nucleotides	not known
RRM1	ribonucleoside-diphosphate reductase	Nucleotides	not known
TYMS	thymidylate synthase	Nucleotides	not known
UMPS	orotate phosphoribosyltransferase,orotidine-5'-phosphate decarboxylase	Pyrimidine Biosynthesis	not known
UQCRB	ubiquinol-6 cytochrome c reductase, Complex III	Oxidative Phosphorylation	not known
UQCRC1	ubiquinol-6 cytochrome c reductase, Complex III	Oxidative Phosphorylation	not known
UQCRC2	ubiquinol-6 cytochrome c reductase, Complex III	Oxidative Phosphorylation	not known
UQCRFS1	ubiquinol-6 cytochrome c reductase, Complex III	Oxidative Phosphorylation	not known
UQCRH	ubiquinol-6 cytochrome c reductase, Complex III	Oxidative Phosphorylation	not known

CAD	aspartate carbamoyltransferase, carbamoyl-phosphate synthase, dihydroorotase	Pyrimidine Biosyn-thesis	not known		
GMPS	GMP synthase	Nucleotides	not known		
PAH	L-Phenylalanine,tetrahydrobiopterin:oxygen oxidoreductase	Tyr, Phe, Trp Biosynthesis	no	Homozygous mice with dysrup-tions in the gene are viable	
TM7SF2	C-14 sterol reductase	Cholesterol Metabo-lism	no	Although a mixture of muta-tions can be lethal, homozy-gotes for mutations are viable	
Gpam	glycerol-3-phosphate acyltransferase	Triacylglycerol Syn-thesis	no	Homozygous mice are viable	12417724
